# Enhanced production of poly-γ-glutamic acid via optimizing the expression cassette of *Vitreoscilla* hemoglobin in *Bacillus licheniformis*

**DOI:** 10.1016/j.synbio.2022.01.006

**Published:** 2022-01-27

**Authors:** Qing Zhang, Yaozhong Chen, Lin Gao, Jian'gang Chen, Xin Ma, Dongbo Cai, Dong Wang, Shouwen Chen

**Affiliations:** a State Key Laboratory of Biocatalysis and Enzyme Engineering, Environmental Microbial Technology Center of Hubei Province, College of Life Sciences, Hubei University, Wuhan, 430062, PR China; b Wuhan Junan Biotechnology Co. Ltd., Wuhan, China; c Tobacco Research Institute, Chinese Academy of Agricultural Sciences, Qingdao, China

**Keywords:** *Vitreoscilla* hemoglobin, Expression element, Twin-arginine translocation, Poly-γ-glutamic acid, *Bacillus licheniformis*

## Abstract

Poly-γ-glutamic acid (γ-PGA) is a natural polymer with various applications, and its high-viscosity hinders oxygen transmission and improvement of synthesis level. *Vitreoscilla* hemoglobin (VHB) has been introduced into various hosts as oxygen carrier, however, its expression strength and contact efficiency with oxygen hindered efficient oxygen transfer and metabolite synthesis. Here, we want to optimize the expression cassette of VHB for γ-PGA production. Firstly, our results implied that γ-PGA yields were enhanced when introducing twin-arginine translocation (Tat) signal peptides (SP_YwbN_, SP_PhoD_ and SP_TorA_) into VHB expression cassette, and the best performance was attained by SP_YwbN_ from *Bacillus subtilis*, the γ-PGA yield of which was 18.53% higher than that of control strain, and intracellular ATP content and oxygen transfer coefficient (K_L_a) were increased by 29.71% and 73.12%, respectively, indicating that VHB mediated by SP_YwbN_ benefited oxygen transfer and ATP generation for γ-PGA synthesis. Furthermore, four promoters were screened, and P_*vgb*_ was proven as the more suitable promoter for VHB expression and γ-PGA synthesis, and γ-PGA yield of attaining strain WX/pP*vgb*-YwbN-Vgb was further increased to 40.59 g/L by 10.18%. Finally, WX/pP*vgb*-YwbN-Vgb was cultivated in 3 L fermentor for fed-batch fermentation, and 46.39 g/L γ-PGA was attained by glucose feeding, increased by 49.26% compared with the initial yield (31.01 g/L). Taken together, this study has attained an efficient VHB expression cassette for oxygen transfer and γ-PGA synthesis, which could also be applied in the production of other metabolites.

## 1 Introduction

High-energy phosphate compounds are universal energy carriers that participate in the whole process of cell life. Acting as the energy currency of cell, adenosine-5′-triphosphate (ATP) affects cell physiological state from various aspects, including cell growth, maintenance metabolism, enzyme activity, metabolites synthesis [1]. With the development of molecular biology and metabolic engineering, various metabolic engineering strategies have emerged to strengthen energy supply for metabolite synthesis, and regulating intracellular ATP supply is becoming an ideal strategy for efficient production of target product (high yield, conversion rate and productivity) [2].

Poly-γ-glutamic acid (γ-PGA) is a natural polymer composed of d-glutamic acid and l-glutamic acid, which was mainly synthesized by *Bacillus*. Because of its excellent properties such as water retention, slow release, and heavy metal chelation, γ-PGA has the wide application prospects in the fields of agriculture, cosmetics, food, medicine, etc [3]. Recently, with the in-depth study of γ-PGA synthetic pathways and regulatory mechanism, various strategies of metabolic engineering has been conducted to improve γ-PGA yield, including strengthening substrate utilization [4,5], introducing exogenous glutamate dehydrogenase from *Corynebacterium glutamicum* [6], deleting or overexpressing γ-PGA hydrolase genes [7,8], strengthening cofactor regeneration [9] and ATP supply [2]. Meanwhile, the γ-PGA yield of 21.3 g/L was produced by *C. glutamicum* after introducing γ-PGA synthase cluster from *B. licheniformis* and systematic metabolic engineering [10]. Due to the high viscosity of γ-PGA, especially at the later stage of fermentation, insufficient oxygen supply might be an important factor restricting ATP supply and γ-PGA synthesis, since the conversion of glutamic acid to γ-PGA requires ATP as cofactor. Previously, several approaches have been conducted to improve ATP supply for γ-PGA synthesis. For instance, nitrate was added into γ-PGA production medium as electron acceptor, which led to 2.3-fold increases of γ-PGA yield [11]. The respiratory chain, nitrate metabolism, ATP synthetic pathways were engineered and *Vitreoscilla* hemoglobin (VHB) was introduced to improve ATP supply and γ-PGA synthesis in *B. licheniformis* [2]. Among these above strategies, introduction of VHB was regarded as the universal strategy to strengthen oxygen transmission and ATP supply.

VHB is the first bacterial hemoglobin discovered by humans, and it showed high homology with eukaryotic hemoglobin. Importantly, VHB has a higher oxygen dissociation rate, which can reversibly combine with oxygen molecule, increase the level of dissolved oxygen and intracellular ATP content. Until now, VHB has been successfully introduced into various hosts (*E. coli*, *Pseudomonas*, *Bacillus*, *Streptococcus zooepidemicus*, *Halomonas*, etc.) to improve cell growth characteristics, strengthen ATP supply, promote metabolite accumulations [[12], [13], [14], [15], [16]]. Zhang et al. have integrated VHB expression cassette into the genome of *Bacillus amyloliquefaciens*, and led to the 7.9% increase of cell biomass and 30% increase of γ-PGA yield [14]. However, due to the difficult contact between oxygen with VHB and inappropriate promoter, the transfer efficiency of oxygen is limited, which affects target product production. Recently, in order to improve the contact efficiency of VHB with oxygen, VHB was expressed and localized in periplasmic space using twin-arginine translocase (Tat) system, which significantly improved the yield of PHB in *Halomonas* [16]*.* Compared to *Halomonas*, gram-positive *Bacillus* owned thicker cell walls, narrower periplasmic space, and unique outer membrane, and no reports has been focused on the optimization of VHB expression cassette for metabolite production, although *Bacillus* is an important industrial production strain.

*Bacillus licheniformis* WX-02 is generally recognized as a safe (GRAS) strain with high-level production of γ-PGA [17], and various metabolic engineering approaches have been conducted to improve γ-PGA yields in our previous researches [2,4]. Here, different signal peptides and promoters were applied to optimize VHB expression and γ-PGA synthesis, and dissolved oxygen content, oxygen transfer coefficient K_L_a, ATP content, as well as γ-PGA yield was measured to analyze the effect of VHB expression cassette optimization on γ-PGA synthesis. Taken together, this research provided an efficient VHB expression cassette for γ-PGA production, which might also be suitable for the syntheses of other metabolites.

## 2 Materials and methods

### 2.1 Strains, plasmids and cultivation conditions

The strains and plasmids used in this study were provided in Table 1. *B. licheniformis* WX-02 (CCTCC M20806) was used as the original strain [17], and VHB expression vectors were constructed basing on the shuttle vector pHY300PLK. The primers used for strain construction and RT-qPCR were provided in Table S1 (Seeing in the Supplementary Materials).

**Table 1:** The strains and plasmids used in this research.

Strains/Plasmids	Description	Resource
*B. licheniformis* WX-02	CCTCC M208065	CCTCC
WX-02/pHY300	WX-02 harboring plasmid pHY300	This study
WX-02/pP43-Vgb	WX-02 harboring plasmid pP43-Vgb	This study
WX-02/pP43-YwbN-Vgb	WX-02 harboring plasmid pP43-SPywbN-Vgb	This study
WX-02/pP43-TorA-Vgb	WX-02 harboring plasmid pP43-SPtorA-Vgb	This study
WX-02/pP43-PhoD-Vgb	WX-02 harboring plasmid pP43-SPphoD-Vgb	This study
WX-02/pP43-SacC-Vgb	WX-02 harboring plasmid pP43-SPSacC-Vgb	This study
WX-02/pP*vgb*-YwbN-Vgb	WX-02 harboring plasmid pP*vgb*-SPywbN-Vgb	This study
WX-02/pPylB-YwbN-Vgb	WX-02 harboring plasmid pPylB-SPywbN-Vgb	This study
WX-02/pPykzA-P43-YwbN-Vgb	WX-02 harboring plasmid pPykzA-P43-SPywbN-Vgb	This study
Plasmids		
pHY300PLK	*E. coli*–*Bacillus* shuttle vector;	Lab collection
pP43-Vgb	pHY300PLK harbors *B. subtilis* P43 promoter, gene *vgb* and *amyL* terminator	This study
pP43-YwbN-Vgb	pHY300PLK harbors P43 promoter, signal peptide of *B. subtilis* YwbN, gene *vgb* and *amyL* terminator	This study
pP43-TorA-Vgb	pHY300PLK harbors P43 promoter, signal peptide of *E. coli* TorA, gene *vgb* and *amyL* terminator	This study
pP43-PhoD-Vgb	pHY300PLK harbors P43 promoter, signal peptide of *B. subtilis* PhoD, gene *vgb* and *amyL* terminator	This study
pP43-SacC-Vgb	pHY300PLK harbors P43 promoter, signal peptide of *B. subtilis* SacC, gene *vgb* and *amyL* terminator	This study
pP*vgb*-YwbN-Vgb	pHY300PLK harbors promoter P_*vgb*_, signal peptide of YwbN, gene *vgb* and *amyL* terminator	This study
pPylB-YwbN-Vgb	pHY300PLK harbors promoter P_ylB_, signal peptide of YwbN, gene *vgb* and *amyL* terminator	This study
pPykzA-P43-YwbN-Vgb	pHY300PLK harboring promoter P_ykzA-P43_, signal peptide of YwbN, gene *vgb* and *amyL* terminator	This study

The basic medium for cell growth is lysogeny broth (LB) medium, and 20 μg/mL tetracycline were added into medium when necessary. The medium for γ-PGA production was 80 g/L glucose, 30 g/L sodium glutamate, 10 g/L sodium citrate, 10 g/L NaNO_3_, 8 g/L NH_4_Cl, 1 g/L K_2_HPO_4_, 1 g/L MgSO_4_·7H_2_O, 1 g/L ZnSO_4_·7H_2_O, 0.15 g/L MnSO_4_·H_2_O, 1 g/L CaCl_2_, pH was adjusted to 7.2 by 6 mol/L HCl. The seeds were cultivated in 250 mL erlenmeyer flask containing 50 mL LB medium for 12 h, then transferred into γ-PGA production medium at a volume ratio of 3%, and cultivated for 32 h (37 °C, 230 r/min).

### 2.2 Construction of VHB expression strains

The method for constructing VHB expression strain was referred to our previously reported method [9,18], and construction procedure of VHB expression strain harboring promoter P43 and signal peptide SP_SacC_ was served as an example. In brief, promoter P43 (EF473728.1) and signal peptide SP_SacC_ (NP_390581.1) from *B. subtilis* 168, original gene sequence of *vgb* from *Vitreoscilla* (AUZ04849.1), and *amyL* terminator (AKQ71831.1) from *B. licheniformis* WX-02 were amplified by corresponding primers, and fused by Splicing Overlap Extension (SOE)-PCR. The fused fragment was inserted into vector pHY300PLK at restriction enzyme sites *Eco*RI/*Xba*I, diagnostic PCR and DNA sequencing were used to confirm the successful construction of VHB expression plasmid pP43-SacC-Vgb. Then, pP43-SacC-Vgb was electro-transferred into *B. licheniformis* WX-02 to attain VHB expression strain, named as WX/pP43-SacC-Vgb. Similarly, other VHB expression strains were attained by the same method.

### 2.3 Analytic methods

Cell biomass was attained via determining the dry cell weight. For γ-PGA fermentation medium, the broth was adjusted to pH 2.0–3.0, centrifuged at 12000 rpm for 5 min, and cell pellets were harvested and dried at 80 °C. γ-PGA yield was determined by high performance liquid chromatography (HPLC), according to our previously reported method [4]. Glucose and glutamic acid concentrations were detected by SBA-40C bio-analyzer (Academy of science, Shandong, China). The intracellular ATP content was determined by ATP assay Kit (Beyotime Biotechnology, China), according to instruction manual.

### 2.4 Gene transcriptional level analysis

Gene transcriptional levels were analyzed basing on the previously reported method [18]. Total RNA was extracted by TRIzol® Reagent, trace DNA was digested by DNase I, and the first stand of cDNA was amplified by Revert Aid First Strand cDNA Synthesis Kit (Thermo, USA). 16S rRNA from *B. licheniformis* WX-02 was served as the reference gene. Gene transcriptional levels of recombinant strains were compared with those of control strain after being normalized to reference gene *16S rRNA*.

### 2.5 Determination of oxygen transfer rate

For determining the oxygen transfer rate, T&J parallel bioreactor (3 L) was used for strain cultivation, using LB medium. *B. licheniformis* was transferred into 1.8 L fermentation medium, and inoculation ratio was 3%, and stirring paddle speed was set as 400 r/min. The dissolved oxygen content was monitored by online DO electrodes, the volume fractions of O_2_ and CO_2_ in exhaust were determined by exhaust gas analyzer, and oxygen transfer coefficient (K_L_a) was calculated by exhaust gas analysis system (T&J Bio-engineering (Shanghai) Co., LTD).

### 2.6 γ-PGA production in 3 L fermenter

For γ-PGA production in 3 L fermenter, *B. licheniformis* was cultivated in 1.8 L γ-PGA production medium. The stirring paddle speed of initial fermentation (0–6 h) was set as 400 r/min, and then increased to 500 rpm until the end of fermentation. In batch culture, the medium for γ-PGA production was the same as that in flask. For fed-batch fermentation, the initial glucose concentration was 60 g/L, when the glucose concentration was lower than 5 g/L, 30 mL 60% glucose was added in the fermentor, and the total volume of additional glucose was 120 mL. The control strategies showed no difference for batch and fed-batch fermentation.

### 2.7 Statistical analyses

At least three parallels were conducted for each experiment, Origin 8.5 and SPSS 18.0 were used for data processing and analysis.

## 3 Results

### 3.1 Effects of different signal peptides on γ-PGA production

Signal peptide plays an important role in protein transport and secretion, and signal peptide optimization is also considered as an effective strategy for efficient production of heterologous proteins [19]. According to the characteristics of sequence and secretion pathway, signal peptides can be divided into the following three types, general Sec, Twin-arginine (Tat) and ABC transporter systems [20]. Here, three Tat-type signal peptides (SP_YwbN_ from *B. subtilis* 168, SP_PhoD_ from *B. licheniformis* WX-02, SP_TorA_ from *E. coli* DH5α) [21], and one Sec-type signal peptide (SP_SacC_ from *B. subtilis* 168) [22] were applied for VHB expression, and the generally recognized P43 promoter was taken as the original promoter [23], attaining VHB expression strains WX/pP43-SacC-Vgb, WX/pP43-PhoD-Vgb, WX/pP43-TorA-Vgb and WX/pP43-YwbN-Vgb, respectively. In addition, the intracellular VHB expression strain WX/pP43-Vgb (Without signal peptide) was attained as control strain.

**Fig. 1:**
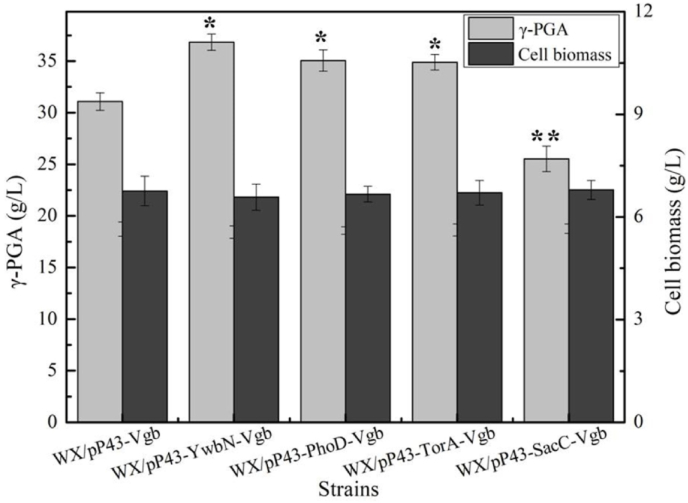
Effects of VHB expression cassettes mediated by different signal peptides (SP_YwbN_, SP_PhoD_, SP_TorA_ and SP_SacC_) on γ-PGA production. Data are represented as the means of three replicates and bars represent the standard deviations, ∗, P < 0.05; and ∗∗, P < 0.01 indicate the significance levels between recombinant strains and control strain.

These recombinant strains were cultivated in γ-PGA production medium, as well as control strain WX/pP43-Vgb. Based on the results of Fig. 1, WX/pP43-Vgb produced 31.08 g/L γ-PGA, γ-PGA yields of 36.84 g/L, 35.05 g/L, 34.89 g/L and 25.53 g/L were attained by WX/pP43-YwbN-Vgb, WX/pP43-PhoD-Vgb, WX/pP43-TorA-Vgb and WX/pP43-SacC-Vgb, respectively. WX/pP43-YwbN-Vgb owned the highest γ-PGA yield, increased by 18.53%, and 17.86% decrease of γ-PGA yield was attained by WX/pP43-SacC-Vgb, compared to control strain WX/pP43-Vgb.

**Fig. 2:**
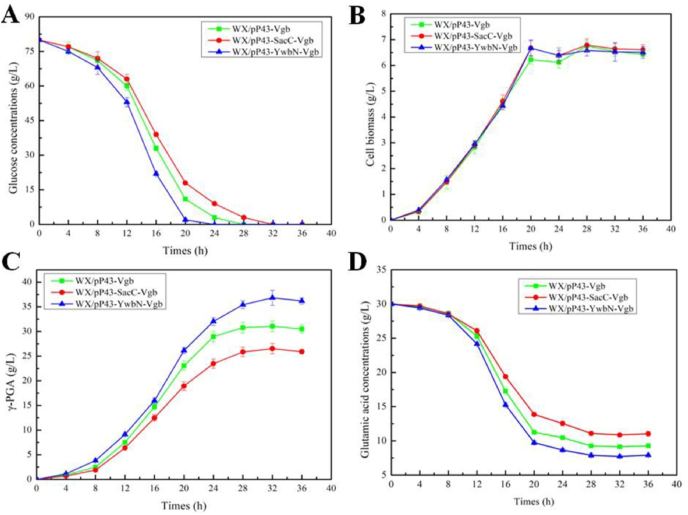
The fermentation process curves of strains WX/pP43-Vgb, WX/pP43-SacC-Vgb and WX/pP43-YwbN-Vgb. A: Glucose concentration, **B:** Cell biomass, **C:** γ-PGA yield, **D:** Glutamic acid concentration.

Furthermore, the cell biomasses, residue glucose, glutamic acid concentrations, and γ-PGA yields of WX/pP43-YwbN-Vgb, WX/pP43-SacC-Vgb and WX/pP43-Vgb were measured during γ-PGA fermentation. Based on the results of Fig. 2, cell biomasses showed no significant difference among these strains, faster glucose and glutamic acid consumption rates were attained by WX/pP43-YwbN-Vgb, and γ-PGA yields of WX/pP43-YwbN-Vgb were higher than those of WX/pP43-Vgb during the whole fermentation process, meanwhile, introduction of SP_SacC_ had the negative effect on γ-PGA production. Since SP_YwbN_, SP_PhoD_ and SP_TorA_ are recognized as Tat-type signal peptides [21], which initially secreted to the folded protein into periplasmic space, and Sec-Type SP_SacC_ mainly secreted the unfolded protein outside cell [24]. Therefore, our results indicated that periplasmic VHB expression using Tat-type signal peptide was beneficial for γ-PGA production, compared to intracellular VHB expression and Sec system.

### 3.2 Effects of VHB expression mediated by different signal peptides on oxygen transfer efficiency

**Fig. 3:**
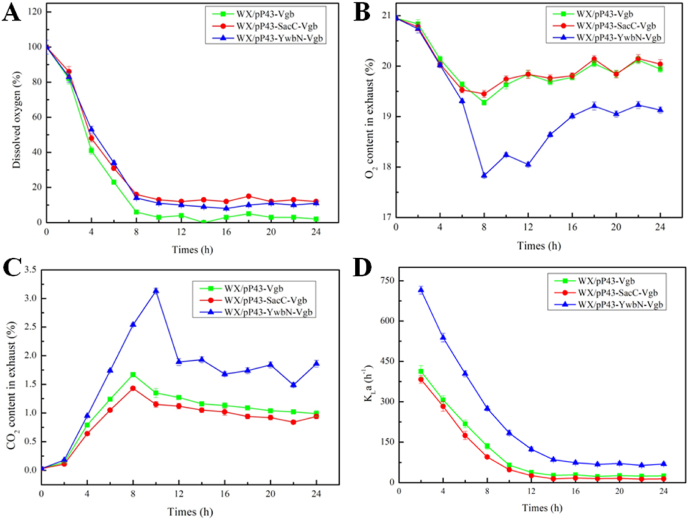
Effects of different signal peptides (SP_YwbN_ and SP_SacC_) on the oxygen transfer efficiency. **A:** Dissolved oxygen, **B:** O_2_ content in exhaust, **C:** CO_2_ content in exhaust., **D:** K_L_a.

**Fig. 4:**
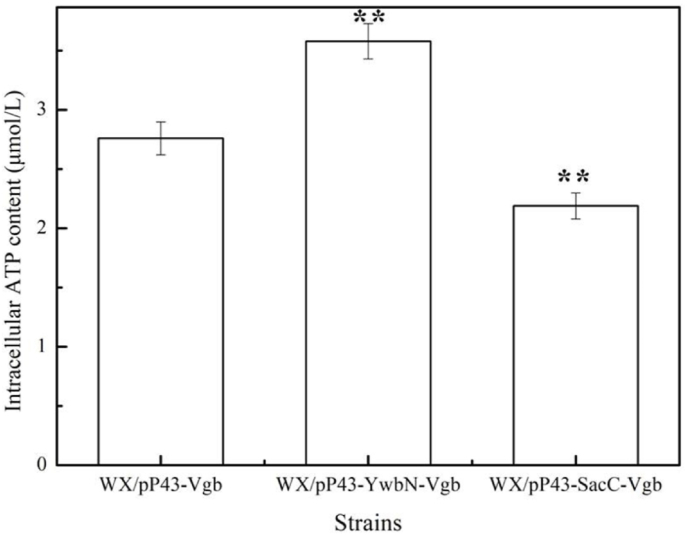
The intracellular ATP contents of WX/pP43-Vgb, WX/pP43-SacC-Vgb and WX/pP43-YwbN-Vgb. Data are represented as the means of three replicates and bars represent the standard deviations, ∗, P < 0.05; and ∗∗, P < 0.01 indicate the significance levels between recombinant strains and control strain.

In order to evaluate the effects of VHB localization and expression on oxygen transfer efficiency, WX/pP43-YwbN-Vgb, WX/pP43-SacC-Vgb and WX/pP43-Vgb were cultivated in 3 L fermenters with LB medium, as the high viscosity of γ-PGA fermentation broth was not conducive to data monitor. Based on the results of Fig. 3, introduction of signal peptides (SP_YwbN_ and SP_SacC_) benefited the increases of dissolved oxygen contents, lower O_2_ and higher CO_2_ contents were detected in the exhaust of WX/pP43-YwbN-Vgb, indicating the increases of oxygen transfer rate (OTR) and carbon dioxide emancipation rate (CER). The maximum K_L_a of WX/pP43-YwbN-Vgb reached 715 h^−1^, increased by 73.12% compared to WX/pP43-Vgb (413 h^−1^). However, lower K_L_a was attained by WX/pP43-SacC-Vgb, indicated that introducing Sec-type SP_SacC_ just increased dissolved oxygen content, and oxygen transfer efficiency was decreased, due to the large amount of oxygen were combined with secreted VHB in fermentation broth [22]. Moreover, intracellular ATP contents of these strains were measured, and the maximum ATP content was 3.58 μmol/L in WX/pP43-YwbN-Vgb, increased by 29.71% compared to WX/pP43-Vgb (2.76 μmol/L), in addition, ATP content of WX/pP43-SacC-Vgb was decreased by 20.65% (Fig. 4), which positively correlated with γ-PGA yields and oxygen transfer efficiency. Therefore, our results demonstrated that VHB expression mediated by Tat-system benefited oxygen transfer, which improved cell respiration for ATP generation and γ-PGA synthesis.

### 3.3 Optimization of the promoters of VHB expression cassette for γ-PGA production

**Fig. 5:**
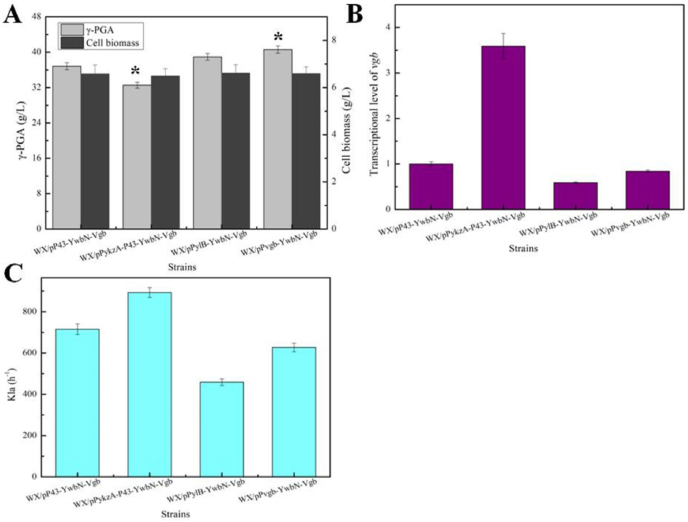
Effects of different promoters on VHB expression and γ-PGA production. A: γ-PGA yield, **B:** Transcriptional levels of gene *vgb*, **C:** K_L_a.

Furthermore, different promoters (dual promoter P_yzkA-P43_ attained by our previous research [25], stationary phase promoter P_ylB_ [26], gene *vgb* native promoter P_*vgb*_ [27]) were applied for VHB expression and γ-PGA production, basing on strain WX/pP43-YwbN-Vgb, attaining recombinant strains WX/pPykzA-P43-YwbN-Vgb, WX/pPylB-YwbN-Vgb and WX/pP*vgb*-YwbN-Vgb, respectively. Among these strains, the highest γ-PGA yield was attained by WX/pP*vgb*-YwbN-Vgb (40.59 g/L), increased by 10.18% compared to WX/pP43-YwbN-Vgb. Meanwhile, our results demonstrated that γ-PGA yield was not enhanced along with the increase of *vgb* transcriptional level, as the imbalance of cell metabolism caused by excessive VHB expression, meanwhile, low expression level of VHB is not conducive to oxygen transfer, which in turn hindered γ-PGA synthesis. In addition, K_L_as of these strains were determined in these strains, which were consistent with VHB expression levels (Fig. 5).

### 3.4 Microbial production of γ-PGA by WX/pP*vgb*-YwbN-Vgb in 3 L fermenter

**Fig. 6:**
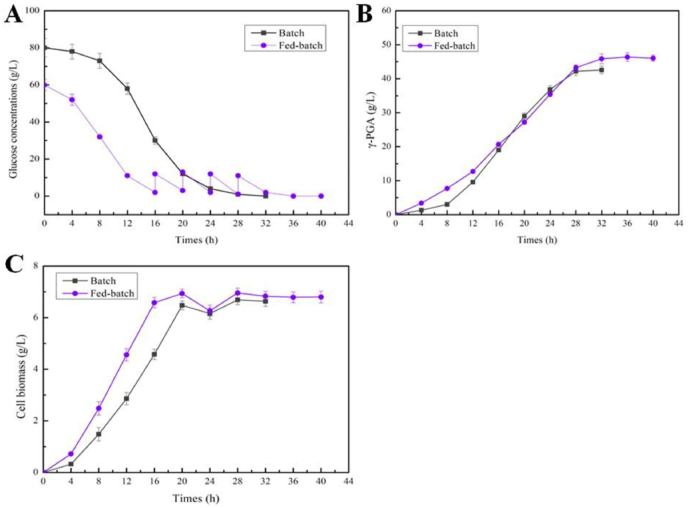
Microbial production of γ-PGA by batch and fed-batch fermentation. A: Glucose concentration, **B:** Cell biomass, **C:** γ-PGA yield.

The batch and fed-batch fermentation were conducted in 3 L fermenters, for microbial production of γ-PGA by WX/pP*vgb*-YwbN-Vgb. In batch culture, WX/pP*vgb*-YwbN-Vgb grew rapidly after the lag period (8 h), glucose was consumed up at 28 h, and the highest yield of γ-PGA reached 42.58 g/L. In fed-culture fermentation, the lag period was significantly shortened (4 h), due to the low initial glucose concentration, and the maximum cell biomasses showed no significant difference between batch and fed-batch fermentation. Glucose was started to feed into the medium at 16 h, and a total of 40 g/L glucose was fed throughout the whole fermentation process, and the maximum γ-PGA yield reached 46.39 g/L, increased by 49.26% compared to the initial yield (Fig. 6).

## 4 Discussions

γ-PGA is an important product with many applications [28], and energy supply served as the critical role in metabolite synthesis [29]. Due to the high viscosity of γ-PGA, oxygen transfer was hindered, which was not conducive to ATP generation and γ-PGA production [11]. VHB is an efficient oxygen transportation carrier, which has been widely introduced into microorganism for efficient oxygen supply and metabolite production. In this study, VHB expression cassette was optimized, including signal peptide and promoter, and fed-batch fermentation was further established for enhanced production of γ-PGA. Our results demonstrated that periplasmic VHB expression benefited oxygen transfer, ATP content and γ-PGA yield were significant enhanced in VHB expression strain meditated by Tat-type signal peptide. Our research provided an effective VHB expression cassette for efficient oxygen transfer and γ-PGA production.

For aerobic microbial fermentation, sufficient oxygen served as the key role in ATP generation and target product synthesis, and this is the dilemma of high-viscosity products. Previously, nitrate replaced oxygen as the electron acceptor in B. licheniformis, ATP content and γ-PGA yield were significant increased in the medium with nitrate addition [11], in addition, other metabolic pathways were also engineered to improve ATP supply for γ-PGA production [2,18]. Acting as an efficient oxygen carrier, VHB has been introduced into various hosts to improve oxygen transfer and metabolic production. However, due to the barrier effect of cell wall and membrane, the contact efficiency of intracellular VHB and oxygen is limited, which is not conducive to efficient oxygen transfer [16]. Here, different types of signal peptides were applied, and VHB expression mediated by Tat-type SP_YwbN_ owned the best performance. In addition, oxygen transfer efficiency and intracellular ATP content were enhanced in VHB strain mediating by SP_YwbN_, indicated that VHB expression mediated by Tat-type SP_YwbN_ enhanced oxygen transfer and ATP supply, which benefited γ-PGA synthesis.

Generally, the secreted proteins transported to extra-cytoplasmic through the special secretory channel. The Sec and Tat pathways are two major transport systems for protein translocation in *Bacillus*, among which, most of secreted proteins are transported through Sec system, and Tat pathway is quite restricted. Tat-system only accepts and translocates the fully folded proteins across cell membrane, and target protein must harbor an N-terminal signal peptide that carries canonical twin-arginine motif. In this research, intracellular VHB expression hindered the contact between oxygen and VHB, and extracellular VHB expression did not allow more oxygen to pass through the cell membrane, both of them were not conducive to oxygen transfer. Tat-type protein shared structural features, including a short N-terminal domain exposed to periplasm or cell wall, and this system has the potential for biopharmaceutical production [30,31]. Through coupling Tat-type signal peptide SP_TorA_ with green fluorescent protein (TorA-GFP), GFP was exported initially to periplasm during batch fermentation, although mostly mature protein (90%) is secreted in the medium eventually, which study offered an efficient approach for producing recombinant protein in *E. coli*, with the potential advantages in terms of purification and downstream processing [32]. In this research, Tat-type SP_YwbN_ guided VHB to localize in periplasmic space, which is not only conducive to oxygen transfer, but also beneficial for cellular respiration metabolism and γ-PGA synthesis, and our results were positively with the previous research [16]. Regrettably, due to the small size of *B. licheniformis*, we failed to verify the VHB localization by ordinary super-resolution electron microscopy. Despite this, our results suggested that the strategy attained by this research might also be effective for other metabolite production, especially for high aerobic fermentation.

With the development of metabolic engineering and synthetic biology, people gradually realized the importance of expression elements in gene expression and metabolic pathway optimization, various transcriptional and post-transcriptional regulatory elements were optimized to regulate gene expression, including promoter [25], 5′-UTR [33], signal peptide [34], terminator [35], etc. Among these elements, promoter was proven as the preferred element for gene expression regulation, as its important role in the initiation of gene transcription [23]. As for protein expression, the strongest promoter generally owned the best performance, reflecting the consistency of gene transcription with protein production [25]. However, this rule is not applicable to metabolite synthesis, with in-depth understanding of metabolic pathways. In recent years, a variety of gradient promoter libraries have been developed to screen the most suitable promoter for target gene expression and product synthesis, their results indicated that the best promoter should be screened at multiple levels, rather than directly applying the strongest one [36]. Based on the results of this research, high-level expression of VHB might cause the cell metabolism imbalance, despite its high oxygen transfer efficiency. VHB expression driven by promoter P43 might be insufficient, resulting in lower oxygen efficiency. In addition, the promoter P_*vgb*_ has been screened by 11 potential low aeration-inducible promoters, which showed the best performance for poly(R-3-hydroxybutyrate) (PHB) synthesis under the micro-oxygen condition [27], suggested that the micro-oxygen condition caused by high viscosity of γ-PGA might also be an important factor in the improvement of P_*vgb*_ performance.

In conclusion, γ-PGA is an important product with various applications. Energy supply served as the critical role in metabolite synthesis. To improve oxygen transfer efficiency during γ-PGA fermentation, expression cassette of VHB was optimized, including signal peptides and promoters. Based on our results, periplasmic VHB expression benefited oxygen transfer, and Tat-type signal peptide SP_YwbN_ and promoter P_*vgb*_ were attained for VHB expression and γ-PGA synthesis. Finally, 46.39 g/L γ-PGA was attained in 3 L fermenter by fed-batch fermentation, increased by 49.26% compared with that of initial yield (31.01 g/L). Taken together, this research provided an efficient expression VHB expression cassette for oxygen efficient transfer, which could also be applied in the production of other metabolites.

## Supplementary information

The additional file is available free of charge in online version of this paper.

## CRediT authorship contribution statement

**Qing Zhang:** Methodology, Investigation, Data curation, Writing – original draft. **Yaozhong Chen:** Methodology, Data curation, Software, Writing – original draft. **Lin Gao:** Investigation, Software. **Jian'gang Chen:** Investigation, Data curation. **Xin Ma:** Writing – review & editing. **Dongbo Cai:** Writing – review & editing. **Dong Wang:** Methodology, Investigation, Data curation, Writing – original draft. **Shouwen Chen:** Data curation, Supervision, Writing – review & editing.

## Declaration of competing interest

The authors declare that they have no competing interests.
