# Supplementary material for: Enhanced production of poly-γ-glutamic acid via optimizing the expression cassette of Vitreoscilla hemoglobin in Bacillus licheniformis
Source: Synth Syst Biotechnol. 2022 Jan 27;7(1):567–73. doi: 10.1016/j.synbio.2022.01.006 (PMC8801620; doi:10.1016/j.synbio.2022.01.006)
Supplement: Multimedia component 1 [file mmc1.docx]

**Enhanced production of poly-γ-glutamic acid production via optimizing expression cassette of *Vitreoscilla* hemoglobin in *Bacillus licheniformis***

Qing Zhang ^a^, Yaozhong Chen ^a^, Lin Gao ^a, c^, Jian’gang Chen ^b^, Xin Ma ^a^, Dongbo Cai ^a^, Dong Wang ^a*^, Shouwen Chen ^a*^

*^a^* *State Key Laboratory of Biocatalysis and Enzyme Engineering*, *Environmental Microbial Technology Center of Hubei Province, College of Life Sciences, Hubei University, Wuhan, 430062, PR China*

^b^ *Wuhan Junan Biotechnology Co., Ltd., Wuhan, China*

*^c^ Tobacco Research Institute, Chinese Academy of Agricultural Sciences*, *Qingdao*, *China*

**Corresponding author*: Prof. Shouwen Chen and Dr. Dong Wang

*Tel./fax.*: +86 027-88666081.

*E-mail address*: [mel212@126.com](mailto:mel212@126.com) (S. Chen) and wdok@sina.cn (D. Wang)

*Postal address*: 368 Youyi Avenue, Wuchang District, Wuhan 430062, Hubei, PR China

**Table S1 Primers used for PCR in this study**

| Primer name | Sequence (5’to 3’) |
| --- | --- |
| 300-T5-F | GAATTCCTGTTATAAAAAAAGGATC |
| 300-T5-R | TCTAGAAGCTTGGGCAAAGCGTTTT |
| P43-vgb-F1 | TTTTTATAACAGGAATTCTGATAGGTGGTATGTTTTCG |
| P43-vgb-R1 | GTTTGCTGGTCTAAGCCCATGTGTACATTCCTCTCTTA |
| P43-vgb-F2 | TAAGAGAGGAATGTACACATGGGCTTAGACCAGCAAAC |
| P43-vgb-R2 | TCCGTCCTCTCTGCTCTTTTATTCAACCGCTTGAGCG |
| P43-vgb-F3 | CGCTCAAGCGGTTGAATAAAAGAGCAGAGAGGACGGA |
| P43-vgb-R3 | TTGCCCAAGCTTCTAGACGCAATAATGCCGTCGCA |
| G-P43-F | GTGTACATTCCTCTCTTACC |
| G-vgb-R | ATGGGCTTAGACCAGCAAACCATTAAC |
| YwbN-F | GAGAGGAATGTACACATGAGCGATGAACAAAAA |
| YwbN-R | GTTTGCTGGTCTAAGCCCATCGCAGTCTGAACAAGCGG |
| PhoD-F | GAGAGGAATGTACACATGAAAAAACTGAGCGAG |
| PhoD-R | GTTTGCTGGTCTAAGCCCATTGCATTGACTTCCATTGC |
| SacC-F | GAGAGGAATGTACACATGAAAAAGAGACTGATT |
| SacC-R | GTTTGCTGGTCTAAGCCCATTGCATCTGCCGAAAATGC |
| TorA-F | GAGAGGAATGTACACATGAACAATAACGATCTC |
| TorA-R | GTTTGCTGGTCTAAGCCCATCGCTTGCGCCGCAGTCGC |
| G-SPywbN-R | ATGAGCGATGAACAAAAAAAGC |
| PylB-F | GAGAGGAATGTACACCATCGTCGAACGCGCTCC |
| PylB-R | TTTTTGTTCATCGCTCATACAAATCTCCCCCTTTGT |
| PykzA-P43-F | GAGAGGAATGTACACGAAATATTGATGTGACAC |
| PykzA-P43-R | TTTTTGTTCATCGCTCATTGATCCTTCCTCCTTTAG |
| Pvgb-F | GAGAGGAATGTACACGAGCGACCTGCGGTGTTT |
| Pvgb-R | TTTTTGTTCATCGCTCATGAGGGTCTTCCTTAAGTT |
| pHY-F | GTTTATTATC CATACCCTTAC |
| pHY-R | CAGATTTCGTGATGCTTGTC |
| 300-T5-P43-F | GAATTCCTGTTATAAAAAAAGGATC |
| 300-T5-TamyL-R | TCTAGAAGCTTGGGCAAAGCGTTTT |
| RT-vgb-F | ATTAACATCATCAAAGCCAC |
| RT-vgb-R | ACAATGTTTGACTGCAATTT |
| RT-16S-F | TCAGCTCGTCTCGTGAGAT |
| RT-16S-R | CGATCCGAACTGAGAACAG |
